# The coordination dynamics of social neuromarkers

**DOI:** 10.3389/fnhum.2015.00563

**Published:** 2015-10-20

**Authors:** Emmanuelle Tognoli, J. A. Scott Kelso

**Affiliations:** ^1^Human Brain and Behavior Laboratory, Center for Complex Systems and Brain Sciences, Florida Atlantic UniversityBoca Raton, FL, USA; ^2^Intelligent System Research Centre, Ulster University, Derry ~ LondonderryUK

**Keywords:** social coordination, alpha, mu, phi complex, brain rhythms, coordination dynamics, complexity

## Abstract

Social behavior is a complex integrative function that entails many aspects of the brain’s sensory, cognitive, emotional and movement capacities. Its neural processes are seldom simultaneous but occur according to precise spatiotemporal choreographies, manifested by the coordination of their oscillations within and between brains. Methods with good temporal resolution can help to identify so-called “neuromarkers” of social function and aid in disentangling the dynamical architecture of social brains. In our ongoing research, we have used dual-electroencephalography (EEG) to study neuromarker dynamics during synchronic interactions in which pairs of subjects coordinate behavior spontaneously and intentionally (social coordination) and during diachronic transactions that require subjects to perceive or behave in turn (action observation, delayed imitation). In this paper, after outlining our dynamical approach to the neurophysiological basis of social behavior, we examine commonalities and differences in the neuromarkers that are recruited for both kinds of tasks. We find the neuromarker landscape to be task-specific: synchronic paradigms of social coordination reveal medial mu, alpha and the phi complex as contributing neuromarkers. Diachronic tasks recruit alpha as well, in addition to lateral mu rhythms and the newly discovered nu and kappa rhythms whose functional significance is still unclear. Social coordination, observation, and delayed imitation share commonality of context: in each of our experiments, subjects exchanged information through visual perception and moved in similar ways. Nonetheless, there was little overlap between their neuromarkers, a result that hints strongly of task-specific neural mechanisms for social behavior. The only neuromarker that transcended both synchronic and diachronic social behaviors was the ubiquitous alpha rhythm, which appears to be a key signature of visually-mediated social behaviors. The present paper is both an entry point and a challenge: much work remains to determine the nature and scope of recruitment of other neuromarkers, and to create theoretical models of their within- and between-brain dynamics during social interaction.

## Introduction

Social neuroscience has garnered tremendous interest over the past decade, as readily appreciated from the large number of dedicated reviews (e.g., Frith and Frith, 2001; Ochsner and Lieberman, 2001; Cacioppo, 2002; Blakemore et al., 2004; Gallese et al., 2004; Insel and Fernald, 2004; Saxe, 2006; Cacioppo et al., 2007; Adolphs, 2009; Behrens et al., 2009; Hari and Kujala, 2009; Schilbach, 2010; Farah, 2011). The entire armamentarium of non-invasive brain imaging methods has been harnessed toward the goal of discovering neural mechanisms of human social behavior, for instance electroencephalography (EEG; Babiloni et al., 2002; Sebanz et al., 2006b; Tognoli et al., 2007a; Lindenberger et al., 2009; De Vico Fallani et al., 2010; Dumas et al., 2010; Thirioux et al., 2010), magnetoencephalography (MEG; Hari et al., 1998), PET (Decety et al., 2002), functional magnetic resonance imaging or functional MRI (fMRI; Iacoboni et al., 1999; Montague et al., 2002; Beauchamp et al., 2003; Olson and Phelps, 2007; Izuma et al., 2008; Saito et al., 2010; Schilbach et al., 2010; Guionnet et al., 2012) and optical imaging (Suda et al., 2010; Funane et al., 2011). However, knowledge of the brain mechanisms involved in social behaviors has tended to lag far behind knowledge of the individual brain. The stakes are high: social behaviors show intricate symptomatic and etiologic ties with a vast number of brain disorders as well as with their treatments (see Table 1). The perspective offered in this review is that many neurophysiological biomarkers (neuromarkers) exist to support distinct aspects of social behavior. We may therefore envision in the future a matrix with each of the conditions of Table 1 having a specific profile of neuromarkers: a trans-nosographic approach. Such a neuromarker profile might help both for diagnosis and for monitoring potential and actual therapies. However, basic discoveries and understanding are much needed before this translational goal can be achieved.

**Table 1:** **Brain conditions affecting social behavior**.

Reference	Domain of investigation
Compton et al. (2005)	Alcohol/drug use disorders
Bennett et al. (2006)	Alzheimer’s disease
Crooks et al. (2008) and Mendez et al. (2005)	Dementia
Segrin (2000), Clark et al. (2003) and Inoue et al. (2006)	Depression
Bassuk et al. (1999), Glei et al. (2005) and Béland et al. (2005)	Ageing
Monetta et al. (2009) and Poletti et al. (2011)	Parkinson’s disease
Mundy et al. (1986), Dawson et al. (2004), Baron-Cohen and Belmonte (2005), Hadjikhani et al. (2006) and Volkmar (2011)	Autism
Giovagnoli et al. (2011)	Epilepsy
Kirsch (2006)	Epilepsy surgery
Genizi et al. (2011)	Benign childhood epilepsy
Yeates et al. (2007)	Children brain disorder
Greenham et al. (2010)	Children brain insults
Lezak and O’Brien (1988) and Gomez-Hernandez et al. (1997)	TBI
Anderson et al. (1999)	Prefrontal lesions
Willis et al. (2010)	Orbitofrontal cortex lesions
Farrant et al. (2005)	Frontal lobe epilepsy
Green and Phillips (2004)	Paranoia
Russell et al. (2000), Couture et al. (2006), Brunet-Gouet and Decety (2006), Schimansky et al. (2010) and Varlet et al. (2012)	Schizophrenia
Jones et al. (2000) and Meyer-Lindenberg et al. (2005)	Williams syndrome

*The long list of clinical conditions in which social behavior is altered suggests that basic discovery of neuromarkers and their functional organization could ultimately have large translational benefits*.

Neuromarkers are important tools to describe the transient and sustained activity of the brain’s functional networks during social behavior. They may appear as oscillatory patterns in electrophysiological measurements due to electrical activity that reverberates in specific brain circuits (Kelso, 1995; Buzsáki, 2006; Kelso and Tognoli, 2007; Tognoli and Kelso, 2009, 2014). Or they may appear as spatial activity patterns in imaging approaches such as fMRI. In the following *(See Sections entitled: “The neuromarker framework: finding local oscillations,” “The neuromarker framework: brain coordination dynamics,” and “The neuromarker framework: functional inferences”)*, we review methodological advances developed in our laboratory and findings that followed from them *(See Sections entitled: “Neuromarker commonalities and differences” and “Toward dynamical models of social brains”)* within the context of experimental paradigms from social coordination dynamics. The dynamical approach is geared toward the analysis and understanding of network-specific oscillatory patterns that are engaged and disengaged during social behavior. The present research aims to elucidate the mapping between dynamic brain patterns and two categorically distinct social functions, namely, synchronic behaviors during which individuals coordinate simultaneously occurring actions; and diachronic behaviors, during which individuals alternate in the perception and production of social behavior *(See Sections entitled: “Synchronic social behaviors” and “Diachronic social behaviors”)*. The emphasis of our approach is on continuous brain recordings rather than the more typical average evoked potentials or average spectra and related measures. Similar efforts are growing quickly in the field of brain-machine interfaces (Guger et al., 2000; Townsend et al., 2004; Kübler et al., 2005; Birbaumer, 2006; Blankertz et al., 2010; Hsu, 2011; Veluvolu et al., 2012), but have yet to be deployed to interpret the dynamics of social behavior. Given the complexity of most social functions, it is likely that multiple routes are available for the realization of particular tasks. This means that to explain social behavior we need to embrace such “degeneracy”—which is what the dynamical neuromarker approach aims to do.

One of the original quests of social neuroscience was toward discovery of “the” neuromarker of social behavior, that is, brain activity emanating from a functional network that transcends social interaction contexts—perhaps in the form of a system of mirror neurons (Gallese et al., 2004; Uddin et al., 2007). However, from many studies it has come to pass that more neuromarkers are recruited and modulated over the course of social behavior than initially presumed. Using EEG to investigate social interactions, our findings reveal that social neuromarkers have oftentimes taken the form of oscillations in the 10 Hz frequency band, a dominant frequency in the cerebral cortex and in cortico-thalamic loops (e.g., Bollimunta et al., 2011). In addition to portraying neuromarkers from this very active region of the EEG spectrum, we will briefly discuss the meaning and relevance of the 10 Hz time scale for social behavior (Note that we use “10 Hz frequency range” as opposed to “alpha range” to describe the band spanning from about 7.5–13 Hz, in order to emphasize that this band contains a variety of potential neuromarkers besides the prominent and well-known alpha rhythm, and to disambiguate ranges and rhythms; see also Bazanova and Vernon, 2014).

Neuromarker multiplicity has led to a number of basic questions about the functional and dynamical architecture of social brains: which major functional system do such neuromarkers support; how do neuromarkers differ from one another; and how do they arise and interact over the course of ordinary social interaction? Questions like these motivate us to propose the methodological framework outlined in Sections “The neuromarker framework: finding local oscillations,” “The neuromarker framework: brain coordination dynamics” and “The neuromarker framework: functional inferences”. Our hope is that revealing the dynamics of neural oscillations will lead to a deeper understanding of the mechanisms underlying social behavior.

An enduring challenge in behavioral, cognitive, affective and social neuroscience is to develop a theory of tasks (Saltzman and Kelso, 1987). This development is especially critical when dealing with dynamical models of the brain, as it may help to infer covert mental processes and determine the timing of their recruitment and dissolution. Today, it seems, we are at a crossroads—having explored a sufficient task repertoire (the *behavior* side of the story) and identified a number of neuromarkers (the *brain* side)—it becomes possible to enquire about the integration of results and their modeling. These are early days in such an enterprise: many elements are still missing and others not yet in definitive place. The present paper is contributed in this spirit. Through methodological advances, systematic experimentation and neurobehavioral theorizing, we attempt to chart a path toward understanding social brains. We end the present review with some ideas on how to cross this frontier in social neuroscience.

## Synchronic Social Behaviors

**Figure 1:**
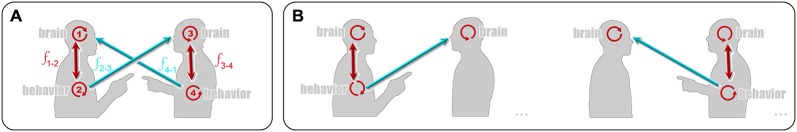
**Task settings.** The flow of information during synchronic **(A)**, and diachronic **(B)** social interactions in a dyadic setting. Circular red arrows describe intrinsic dynamics in neural and behavioral subsystems respectively. Straight red arrows describe movement and perceptual information flows that are circumscribed to an individual; blue arrows represent information flows that cross to the other individual (social coupling). During synchronic social behaviors **(A)**, information flows bidirectionally between all parts of the system. In contrast, during diachronic social behavior **(B)**, only one person acts at a given time and one behavioral subsystem is disengaged. The two vignettes in **(B)** illustrate turns of behavior between the two individuals. See details in text.

Synchronic social behaviors engage simultaneous action and perception processes. Tango dancing, choir singing, driving in traffic, executing shared-tasks such as lifting heavy furniture in tandem or performing surgery are examples of synchronic behaviors, with varying degrees of symmetry between the actions performed and the varying effector and sensory pathways involved in action~perception couplings. In such interactions, information flows continuously and reciprocally between people through perceptual channels (Figure 1A, blue arrows), creating linkages at both brain and behavioral levels.

A unique characteristic of synchronic behavior is that the actions of one individual (e.g., Figure 1A, annotation 2) are readily able to modulate a partner’s behavior (Figure 1A, annotation 4) with information flowing in a reciprocal, bidirectional fashion. Information about self-produced movement is returned back to oneself and is updated based on the actions of one’s partner (Figure 1A, *f*_4–1_). With both partners simultaneously engaged in such informational exchange—each continuously perturbing the other—a system is formed that enters a kind of self-organization that exhibits rich dynamical behavior (Kelso, 1995; Sebanz et al., 2006a; Tognoli et al., 2007a; Oullier et al., 2008; Tognoli, 2008; Oullier and Kelso, 2009; Konvalinka et al., 2010; Riley et al., 2011; Duran and Dale, 2014). To probe this dynamics, the social coordination paradigm assesses the behavioral and neural organization of subjects as they continuously perform simple rhythmic index finger movements (extensions/flexions). Differences between behaviors produced in individual and social contexts are assessed by manipulating the perceptual flow between people, switching vision of each others’ action on and off with the help of an optical barrier (see supplementary materials S1–S2). The advantage of this very basic, canonical situation is that it provides explicit and continuous measures of social coupling through the dynamics of a collective variable, the relative phase (Tognoli et al., 2007a; Oullier et al., 2008; Tognoli, 2008; Oullier and Kelso, 2009; Tognoli et al., 2011), akin to studies of bimanual (e.g., Kelso, 1984; Haken et al., 1985; Swinnen and Wenderoth, 2004; Banerjee et al., 2012), sensorimotor (Kelso et al., 1990; Schmidt et al., 1990; Wimmers et al., 1992) and postural coordination (Varlet et al., 2011). To study the potency of perceptual coupling and corresponding neural correlates during this spontaneous form of social coordination, we further distinguish trials during which subjects entered a state of phase-locked collective behavior from those that did not (Tognoli et al., 2007a).

## Diachronic Social Behaviors

In contrast to synchronic behavior, in diachronic social transactions only one participant acts at a given time. Examples of such behavioral transactions include conversation with well-defined turn-taking, imitating a person’s facial expression or accent, and learning a surgical gesture by observing a demonstrator in medical school. Coupling in the system is ensured by perceptual flows to the observer’s brain (Figure 1B, blue arrows), but there information flow reaches an end-point—at least momentarily until role settings are eventually modified. As a result, information flows do not circulate continuously in the system. If all relevant influences stopped in the immediacy of perceptual exchange, this type of social transaction would seem less useful than its ubiquity suggests. However, it appears that such exchanges rely upon delayed influences—possibly buffered in the observer’s brain through memory processes—and mutual social influences are therefore allowed to resume at slower time scales (see Tognoli and Kelso, 2013 for a theoretical discussion on time scales and causality in complex systems). Experimental tasks that probe such diachronic behaviors include action observation and delayed imitation. In our implementation (Suutari et al., 2010), we instructed pairs of participants to first observe then imitate index finger movements in turn, during two periods of continuous behavior (8 s long) separated by retention, pause and control intervals for individual behaviors (see supplementary materials S3). We studied social neuromarkers and their dynamics when subjects observed their partner’s action, performed an action alone or under the observation of their partner, imitated the action they observed earlier, and during rest.

## The Neuromarker Framework: Finding Local Oscillations

From dual EEG recordings, we examined the repertoire of brainwaves (brain rhythms, periodic and aperiodic oscillations) recruited for social tasks. Brainwaves carry a 3-sided signature of underlying neurophysiological processes: (1) spatial organization (how energy is distributed over the scalp -an indirect manifestation of the originating neural structures); (2) spectral properties (the frequencies at which brainwaves operate—a manifestation of their temporal extent and affordance for interaction with other neuromarkers); and (3) functional dependency (i.e., which behavioral/mental/affective processes modulate them). In other words, analysis of brainwaves addresses the structure, dynamics and function of the brain (e.g., Kelso, 1995; Freeman, 2000; Basar, 2004; Bressler and Tognoli, 2006; Buzsáki, 2006; Kelso and Tognoli, 2007).

**Figure 2:**
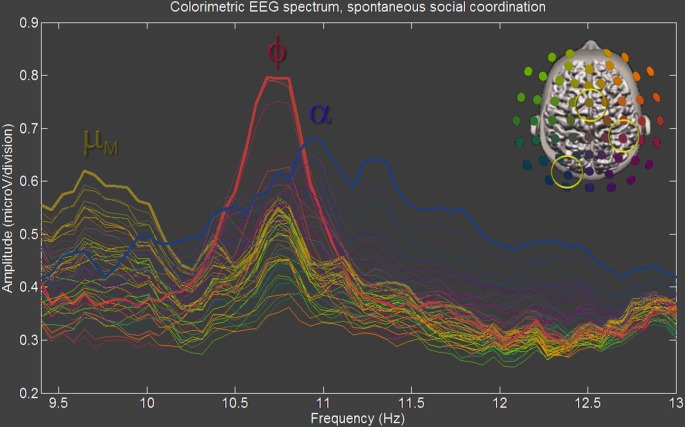
**Parsing neuromarkers.** Neuromarkers can be parsed using multi-electrode spectra with high spectral resolution (here bin size is 0.06 Hz) and colorimetric encoding of spatial organization (following colorimetric legend shown in upper right corner). In this figure adapted from Tognoli et al. (2007a), sampled from a subject performing spontaneous social coordination -a synchronic task- 3 neuromarkers are observed that include mu medial (appearing in brown color as a result of its fronto-central topography), left alpha (blue, left occipital region) and phi (burgundy, right centro-parietal region). Note spectral proximity, especially for phi and alpha. Neuromarkers are quantified by identifying the boundaries of spectral peaks, when power departs from and returns to background power, and by integrating power over all the bins included in this interval (see supplementary materials S4).

Importantly, from the theory of coupled oscillators, it ensues that neural oscillations meant to work together need to operate on similar time scales, or equivalently, frequencies. If the binding/coordination mechanism at play is phase- and frequency-locking or a more subtle metastability (Kelso, 1995, 2012; Tognoli and Kelso, 2009, 2014) this constraint translates into neural ensembles’ operating with similar (or near integer-related) frequencies (see, e.g., deGuzman and Kelso, 1991; Bressler and Kelso, 2001; Bressler and Tognoli, 2006; Palva and Palva, 2007, 2012; Tognoli and Kelso, 2009, 2014; Tass et al., 1999). As a result, spectral overlap is often present, a feature that is poorly accounted for in traditional EEG studies. For example, when examining the 10 Hz band at the usual spectral resolution of ~1 Hz, overlap translates into a blurred spectral and spatial differentiation of neural oscillations. More specifically, one sees an irregular-shaped peak in the spectrum, with its power distribution changing from place to place over the surface of the scalp. This amorphous view conceals a number of discrete peaks each with their own frequency and topography (such as the three peaks shown in Figure 2), but so close that they may merge spatially and spectrally at low resolution. Our framework of brain coordination dynamics rests on high-resolution spectral analysis of EEG with colorimetric encoding of topography- a set of techniques that performs well at distinguishing oscillations with spectral and spatial proximity (Tognoli and Kelso, 2009). When sufficient spatial and spectral resolution are achieved (increasing sensor density to augment spatial resolution and either increasing the amount of continuous time in Fourier analysis or lengthening the time interval artificially using zero padding to augment spectral resolution), crisp regional distributions of power do appear. Using such techniques, it is possible to measure the functional specificity of brain rhythms without the corrupting effect of other oscillations located nearby.

Although the general architecture of human brains may be the same, on a fine grain level every brain circuit is different. Hence, a neuromarker may shift slightly in frequency and topography from one subject to another. Critically, identification of neuromarkers needs to be conducted on a subject by subject basis (see also Veluvolu et al., 2012, for a related account). At this stage, interindividual comparisons are performed on parsed neuromarkers (their conditional power or/and their time course), not on the less refined picture of power distribution that is obtained from grand-average spectra (mean of all individual spectra, which again causes blurring due to spatial and spectral variations between subjects).

## The Neuromarker Framework: Brain Coordination Dynamics

Oscillations may be studied in average spectra (as in Figure 2) and continuous time. We hypothesize that such oscillations reveal the transient activation of unique functional networks in the brain. Under such an hypothesis, it is possible to establish a time-course describing the engagement and disengagement of brain networks. The latter coexist with another timeline of descriptors, namely one that refers to the brain’s functional organization at the level of behavior, perception, cognition and volition *(See Section below entitled: “The neuromarker framework: functional inferences”)*. The challenge for social neuroscience (and for neuroscience in general) is to recognize that both neural and behavioral/cognitive levels may be characterized in terms of their dynamics and that dynamics offers a means by which to relate them (Kelso, 1995, 2012; Buzsáki, 2006).

**Figure 3:**
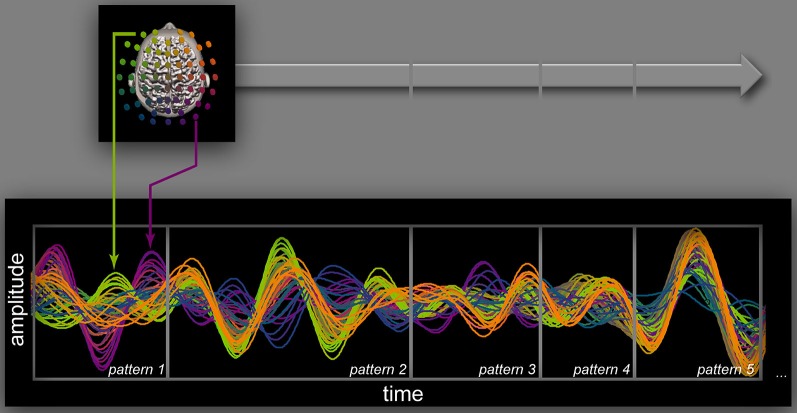
**Brain dynamics.** A sequence of five oscillatory patterns segmented from continuous, band-pass filtered EEG in a synchronic task of intentional social coordination. Filters (7–13.5Hz) were set to retain activity in the 10 Hz range, a prominent feature of human waking EEG. Patterns were segmented manually by two trained examiners who analyzed the spatiotemporal evolution of phase aggregates (Benites et al., 2010). Results were later confirmed using an automatic segmentation algorithm. Each pattern inside the gray frames is best explained by the transient organization of a few macroscopic ensembles that interact through phase-locking or metastability. For instance, the first pattern shows phase aggregates that are suggestive of one gyral and one sulcal source (green and magenta arrows respectively; source estimation provides some indication on their cortical origin). Short-lived configurations tend to succeed one another (e.g., magenta phase aggregate ends with the edge of the first box, giving way to new phase aggregates in the second gray box). Putatively, this organization provides support for ongoing functional processes. Note that such neural organization in the 10 Hz frequency band sustains transient patterns with a typical duration of 1–200 ms, a crucial time-scale for human behavior, both individual and social.

Neuromarker dynamics can be probabilistically approached using wavelet analysis (see, e.g., Tognoli et al., 2007a; Suutari et al., 2010) within the spatio-spectral domain identified from a “static” neuromarker approach (Figure 2). This provides a picture of the brain in which macroscopic ensembles fluctuate smoothly in amplitude over time, an imperfect but heuristic means to explore macroscale neural dynamics. The wavelet approach is heuristic in the sense that following selection of the right electrode and frequency band for a neuromarker of interest, it tends to maximize the correspondence between signal power fluctuations and the genuine time course of a functional process. Fundamentally, the inverse problem prevents one from identifying source dynamics solely on the basis of information from scalp recordings. As a result, electrode-based wavelet approaches (and related methods) are far from perfect. Since a number of distant neural ensembles contribute to the scalp signal in the same scalp neighborhood, there is no guarantee that a unique neural ensemble is tracked continuously by monitoring power at selected electrodes. Rather, electrode power is determined by a number of neural ensembles in turn. A much more precise approach includes segmentation and classification of transient spatiotemporal patterns and analysis of their coordination dynamics (Tognoli and Kelso, 2009; Benites et al., 2010; Fuchs et al., 2010; Tognoli et al., 2011; and Figure 3; see also Lehmann et al., 2006), to be followed by reconstruction of their source dynamics (Pascual-Marqui et al., 2002; Murzin et al., 2011). Such methods provide a picture in which sources are intermittently on and off. As discussed in Tognoli and Kelso (2009), we are less interested in power/amplitude quantifications (which are inappropriate measures of neural source strength in the first place, Tognoli and Kelso, 2009), than with the lifespan of large scale patterns (duration and recurrence) and their dynamical interaction with other neural ensembles (e.g., phase relationships within patterns; vicinity of other patterns that entertain causal precedence and consequence). In our approach, all such dynamical attributes are scrutinized in terms of their possible functional significance.

## The Neuromarker Framework: Functional Inferences

**Figure 4:**
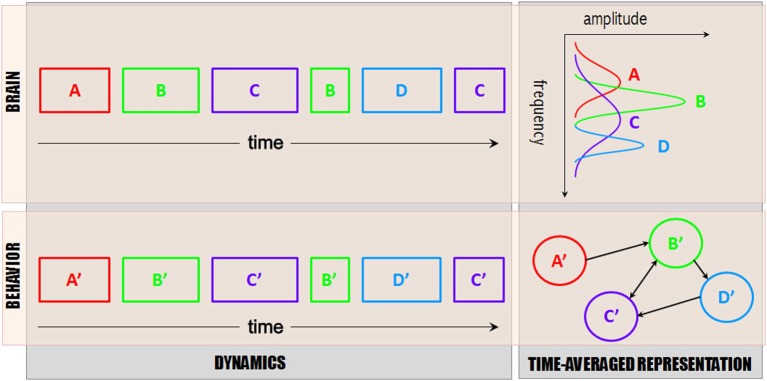
**Brain~behavior scheme.** Dynamical descriptions of brain functional networks (top left) and inferred functional processes (bottom left), along with their time-averaged representation (functional graph on lower right and power spectrum on the upper right (note rotated axes to reflect the fact that amplitude is largely inherited from the cumulative duration of the patterns, along with their frequency consistency over time). For simplicity, only one frequency band is represented (say, 10 Hz), and only one process at a time (i.e., no network interaction). In reality, multiple frequency bands (and associated functional processes) occur at the same time. Typically, networks are co-activated and exhibit transient interactions, e.g., via phase locking and metastability. The goal of functional inference is to identify the functional processes (bottom rectangles) that match spatiotemporal patterns of brain activity (top rectangles) and their temporal footprints, so that correspondences between brain and behavior can be uncovered. Though simplistic, a translational language along these lines would propel our understanding of social brain functions and lead the way toward explanatory models.

Inferences about brain~behavior correspondences (a temporal puzzle, see Figure 4) represent a key challenge that must be overcome in order to achieve adequate explanatory models of social brains. The rich phenomenological language of human behavior and cognition has been developed over centuries of scholarly enquiry, accelerated in recent decades due to the thrust of cognitive (neuro)science. We postulate that the functional language of human behavior (e.g., sociocognitive and affective processes) maps onto discrete neural patterns, i.e., those that can be captured from segmentation of continuous EEG *(See above Section entitled “The neuromarker framework: brain coordination dynamics”)*. Due to the convergent~divergent connectivity of the brain, the mapping is likely to be degenerate: the same output pattern may be produced by a number of different interacting brain structures, and alternative pathways between neural structures are capable of producing functionally equivalent cortical patterns (Edelman and Gally, 2001; Tononi, 2010; Kelso, 2012)—the key signature of self-organized synergies or coordinative structures (Kelso et al., 1984; Kelso, 1995). The empirical challenge then becomes one of matching temporally inferred functional processes and observed brain patterns (Figure 4, left). Such inference is guided by the study of neuromarkers (as in Figure 2), and neuromarker dynamics (as in Figure 3). A sound strategy consists of meta-analyses: after a neuromarker has been revealed through the study of multiple tasks and experimental manipulations, it becomes possible to narrow down its functional significance more precisely, thereby separating its true functional meaning from sporadically co-varying effects.

Difficulties lie in the fact that (1) theories of tasks are seldom based on explicit, observable quantities and (2) such descriptions, despite their ready reduction into serial models, are not grounded in a dynamical framework that allows one to establish unambiguous time addresses for the engagement and disengagement of functional processes. A place to begin such an endeavor is with functional processes that have explicit temporal footprints, as in our social coordination paradigms. Time-averaged neuromarkers (obtained from the methodology spelled out in Section “Synchronic social behaviors”) and their reactivity also provide tractable material that may lead to establishing neuro-functional relationships (see Table 2 below).

**Table 2:** **Neuromarker meta-analysis**.

	Neuromarker	Peak frequency	Topography	Task dependence
Only in synchronic tasks (Tognoli et al., 2007a, b, 2011; Tognoli, 2008)	Mu medial	9.1 Hz (1.1)	FCz	- recruited during spontaneous coordination
				- suppressed during social interaction
				- rebounds during intrinsic, self-produced movement
	Phi complex	10.9 Hz (0.9)	CP4	- phi 1 increases in spontaneous coordination when subjectsdo not synchronize with each other
				- phi 2 increases when subjects synchronize with each other
				- phi complex recruitment and modulation is strongest during intentional social coordination
Both task types	Alpha	9.9 Hz (0.9)	PO7 (left), PO8 (right), POz (midline aggregate)	- increased by drowsiness
				- decreased by visual input
				- decreased further by vision of the partner’s movement (larger decrease than with non-social stimuli)
				- further decreased if partner’s movement is more variable
Only in diachronic tasks(Suutari et al., 2010; Tognoli et al., 2011)	Left and right central mu	Left: 10.6 Hz (0.9) Right: 11.4 Hz (0.8)	C3 (left), and C4 (right)	- left and right mu depressed during self movement; rebound at self-movement arrest
				- no systematical decrease during social observation
				- exhibit dynamic aftereffects (suppression, rebound) atcessation of observed movement
				- right mu vanishes tonically when people memorize observed behavior (right hand movement)
	Nu	10.1 Hz (1.1)	CPz	- decreases during self-movement
				- increases when self-movement is performedin view of another person
	Kappa	11.2 Hz (0.7)	FC2	- tendency to decrease any time either partner performsa movement in view of the other

*Summary of spatial, spectral and functional properties of neuromarkers involved in synchronic and diachronic social behavior (see also Figure 6 for topographical maps and colorimetric spectra). The data presented are group results obtained from the samples of subjects that have participated in our studies. Peak frequency (measured from high-resolution spectra) describes the arithmetic mean of the samples with standard deviation in parenthesis. The electrode reported in column topography refers to the mode (electrodes most frequently observed across subjects that bear largest spectral energy, named according to the 10 percent system, Chatrian et al., 1985). All recordings were performed with linked-mastoid reference. “Task dependence” refers to conditions in which power is modulated, a precursor to inferences about function*.

Descriptions of behavior and cognition are especially fruitful for slower and more global functional processes, the time-scale of which was amenable to observational and experimental tools of earlier times. In contrast, faster processes (timescales of tens of milliseconds and less) have not systematically received distinct names and descriptions. Short-lived patterns that are uniquely tracked with dynamic brain imaging techniques such as EEG and MEG may hold keys to advancing understanding of social behavior (for instance, irrespective of their functional brevity, they may be keys to certain deficits). Identifying causal chains of neuro-functional processes at faster time-scales—not typically available in social cognition/behavior settings—may be one of the most valued advances that social neuroscience can make.

## Neuromarker Commonalities and Differences

**Figure 5:**
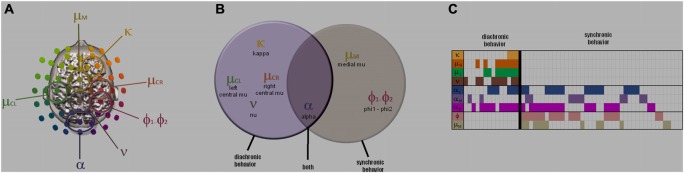
**The neuromarker repertoire.** Overview of neuromarkers contributing to social behavior obtained from meta-analysis of three studies (supplementary materials S1-S3). **(A)** shows their scalp topography, **(B)** a Venn diagram of their recruitment in studies of synchronic and diachronic social behavior, and **(C)** a meta-analytic table of their interindividual occurrence. Neuromarker location in **(A)** indicates sensor carrying highest power on the scalp, keeping in mind that this does not imply regional homology with underlying cortical structures. Each column of **(C)** specifies one of fifty four subjects enrolled in our experiments of social behavior, each row corresponding to a neuromarker. When a neuromarker was detected in a subject, its cell is marked with a color, else it is left blank. Note empty sectors in the lower left and upper right sectors that suggest specific neuromarker landscape for the two types of social behaviors.

**Figure 6:**
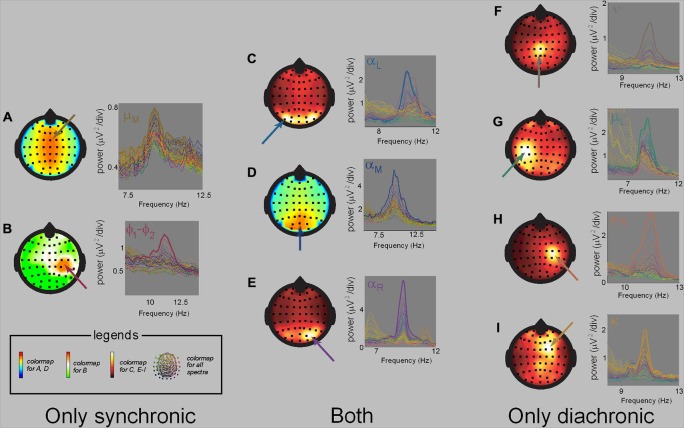
**Task-specific neuromarkers.** Spatial and spectral properties of the neuromarkers referenced in Table 2 (adapted from Tognoli et al., 2007a; Suutari et al., 2010). On the left **(A–B)** are neuromarkers mu medial **(A)** and phi **(B)**, that were only observed in synchronic tasks. On the right are neuromarkers nu **(F)**, left and right mu **(G,H)** and kappa **(I)**, that were only observed in diachronic tasks. **(C–E)** has alpha neuromarkers that were observed in both types of task, and shows the medial form **(D)**, and its lateralized variants, emphasizing the left **(C)** and right hemisphere **(E)**. Neuromarker discovery is aided by the color of their spectra, which are not chosen arbitrarily but inherited from neuromarker’s spatial organization.

The repertoire of neuromarkers observed during our social tasks (synchronic social behaviors of spontaneous and intentional social coordination; diachronic social behaviors of action observation and delayed imitation; Tognoli et al. (2007a,b, 2011); Tognoli (2008) and Suutari et al. (2010); (see also supplementary materials) is summarized in Figure 5. During synchronic social behaviors, a set of neuromarkers was recruited that included the alpha rhythm, the phi complex and especially when interaction was spontaneous, a medial mu rhythm (Tognoli et al., 2007a,b). During diachronic social behaviors, alpha was also observed, but mu medial and the phi complex were not detectable. In addition, left and right central mu appeared as did two newly described nu and kappa rhythms (Suutari et al., 2010). The spatial, spectral and functional properties observed for these rhythms in our samples of subjects are reported in Table 2 and Figure 6. Keeping in mind the high-resolution spectral analysis implemented here, accuracy of estimation is aligned with the spectral resolution of the coarsest dataset, i.e., 0.1 Hz. The data presented in Table 2 are group results obtained from the samples of subjects that have participated in our studies (peak frequency describes the arithmetic mean of the samples; electrode location refers to the mode). Of course, large populations would be helpful to establish robust normative properties of neuromarkers (something that at this time, we forgo in favor of smaller, discovery-based studies). Table 2 summarizes spatial, spectral and functional properties as a starting point to identifying new neuromarkers and with the aim of helping others in the field who share similar goals.

The only neuromarker that transcended both synchronic and diachronic social behavior was the alpha rhythm, a neuromarker associated with visual attention (Mulholland, 1972; Klimesch et al., 1998; Palva and Palva, 2011). All of our studies revealed that vision of the partner substantially reduces alpha power. With its separation of social and self behaviors in distinct experimental phases, our study of action observation further allowed us to show that alpha fluctuated with the complexity of behavioral information acquired about the partner. In Suutari et al. (2010), single trial alpha power was low when observers were exposed to finger movements with high cycle to cycle variance. By contrast, alpha increased with more regular movements. Put another way, the individual brain’s alpha rhythm appears to be a pertinent measuring instrument of the complexity embedded in interpersonal information flows (see also Müller et al., 2003 for related account in non-social visual perception).

A social interaction exists only if social partners acquire information about each other (see blue arrows in Figure 1). Our results suggest that the alpha rhythm is a key neuromarker of visually-mediated social behavior (putatively, social transactions mediated by other sensory channels would have their own signatures, see, e.g., Pineda, 2005 for candidates). Alpha modulation is often overlooked in EEG/MEG studies of social interaction in favor of mu rhythms. We suggest however that alpha’s sensitivity to informational exchange between partners, its large amplitude in human EEG and robust presence in most subjects makes it an important neuromarker of social behavior (see The neuromarker framework: “finding local oscillations” Section for strategies to disambiguate alpha, mu and other spectrally similar neuromarkers). Furthermore, in visual detection tasks, it has been shown that alpha suppression is spatially informative, with attention to the right hemifield depressing specifically left alpha rhythm and vice-versa (Worden et al., 2000; Sauseng et al., 2005). Such lateralization could be useful to disentangle self and social attention in experimental designs that carefully manipulate the spatial arrangement of self and other—with the potential outcome that roles in social interactions could be quantified as a function of the spatial deployment of attentional resources. Moreover, interindividual variation in alpha suppression could reveal the extent of social engagement and task-related social affinities, with consequent applications to a variety of domains relevant to human social behavior.

## Toward Dynamical Models of Social Brains

As we observe many neuromarkers and their intermittent dynamics in dual-EEG recordings (see “The neuromarker framework: brain coordination dynamics” Section), we are led to question their spatiotemporal organization—how the functional processes that participate in social behavior are orchestrated. Until now, at the largest scale of complete dual-EEG experiments, we have achieved either a static neuromarker description (as in “The neuromarker framework: finding local oscillations” Section), or a probabilistic description of their dynamics using wavelet analysis on selected frequency bands and spatial sites (e.g., Tognoli et al., 2007a; Suutari et al., 2010; see discussion in “The neuromarker framework: brain coordination dynamics” Section). Based on theoretical and methodological work (Tognoli and Kelso, 2009), we have also started to study the dynamic patterns of dual-EEG (see Figure 3 and text thereafter) on particularly interesting aspects of social behavior such as the loss or establishment of coordinated action. The first stage of this analysis is a segmentation of continuous (band-selected) EEG. We have implemented either a manual analysis of the oscillations’ phase, frequency and topography (Benites et al., 2010), or an automatic segmentation method examining the eigenvalue tradeoff between two principal modes of the EEG power envelope derived from a rotating wave approximation (Fuchs et al., 2010). The result of both approaches is to parse each participant’s EEG into a sequence of dynamic patterns (see Figure 3). This sequence is then matched to an estimation of the time course of inferred functional processes (Figure 4), with the goal of connecting their dynamics. This framework extends our earlier efforts that found a tight connection between behavioral and neural dynamics once an appropriate space of collective variables was identified. Spatiotemporal measures of brain activity tracked kinematic measures of sensorimotor coordination both empirically (Kelso et al., 1998) and in a theoretical model of the underlying neural field dynamics (Fuchs et al., 2000).

As more and more insights into the function of neuromarkers becomes available, it should become possible to solve the temporal puzzle of brain~behavior as presented in Figure 4. When that point is reached, we will be able to draft dynamical models of social processes at the combined levels of brain and behavior and to study their variation in different situations (e.g., social skill development, disease, effects of pharmacological treatment, etc.).

**Figure 7:**
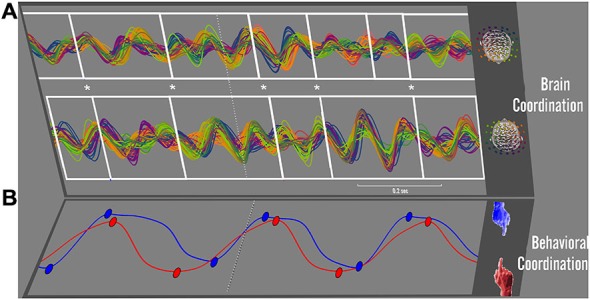
**Brain~behavior coordination.** Synchronized patterns between brains, in a synchronic behavior of intentional social coordination (after Tognoli et al., 2007b). Continuous dual-EEG is shown in the 10 Hz frequency band for a pair of interacting subjects in **(A)**, with electrode signals encoded using the colorimetric legend shown on the right (EEG from one subject on top, the other on the bottom). Changes in spatiotemporal organization of brainwaves were determined by two trained examiners who were blind to the associated behavioral variables (Benites et al., 2010). A manual segmentation was performed separately on each subject’s EEG. Transitions are marked by successive white frames, following the method outlined in Section “The neuromarker framework: brain coordination dynamics” and Figure 3. In this sample trial, subjects were instructed to coordinate finger movements inphase (see red and blue movement trajectories of right index fingers in **B**). The dashed line in **(B)** indicates the moment at which they successfully coordinated their behavior (with the movements’ relative phase exhibiting a sudden phase transition to inphase, not shown). The entire temporal window displayed is about 1 s long and relates to the intentional transition process from independent to coordinated behavior. In this window, the transition between subjects’ brain patterns reveals strong tendencies for coincidence (see series of asterisks in **(A)**, cueing temporal proximity of each subject’s brain pattern transitions). Note that the dynamic patterns of each participant’s brain activity have distinct spatial, spectral, and phase organization. Neural transitions are coupled, but not the spatiotemporal neural patterns located between them.

In the preceding, we have examined collective behavior and its relation to brain activity, but only a single brain at a time. With social neuroscience born from cognitive neuroscience, there is a temptation to segregate the neural activity of participants to fit the existing framework of single-brain neuroscience. A true social neuroscience, however, will only realize itself when it fully integrates neural activity of every participant in a common analysis scheme. Efforts to do so have been undertaken by collecting synced records of brain activity from multiple people (e.g., dual-EEG: Tognoli et al., 2007a; or fMRI hyperscanning: Montague et al., 2002) and by formulating novel analysis frameworks that combine the neural dynamics from multiple subjects (Lindenberger et al., 2009; Dumas et al., 2010; Dodel et al., 2011; Tognoli et al., 2011). With brains chock full of oscillations that are coupled between people through inter-personal perceptual flows, a straightforward hypothesis is that oscillations enter collective states of phase-locking and frequency coupling between the brains of interacting partners—a hypothesis that has been pursued by ourselves and others (e.g., Lindenberger et al., 2009; Dumas et al., 2010; see also Funane et al., 2011; for related hemodynamic account). Our research has yet to uncover unambiguous evidence of phase-locking between the brains of people as they engage in social behavior. Moreover, our longstanding theoretical inclination is toward metastable coordination dynamics, where tendencies for integration coexist with tendencies for segregation (e.g., Kelso, 1995; Kelso and Tognoli, 2007; Tognoli and Kelso, 2009, 2014). The reason we suspect that phase synchrony is seldom observed is that at the level of dynamic patterns (and in the frequency bands examined, especially around 10 Hz), limited symmetry exists between the instantaneous networks formed in each person’s brain (see example Figure 7). However, in applying the aforementioned segmentation methods to social coordination tasks, we encountered evidence of another, less expected mechanism of coupling between brains (Benites et al., 2010; Fuchs et al., 2010). On the one hand, each subject’s neuro-functional activity was distinct (compare upper and lower white frames in Figure 7A, and note patterns’ lack of correspondence in topography, frequency and phase), yet on the other hand, the moment at which those patterns changed in each partner coincided (note temporal coincidence of white frames’ edges marked with asterisks in Figure 7). In other words, it was not the oscillatory neural activity proper that was synchronized between people but rather the underlying temporal structure of their recruitment and dissolution. An analogy to such inter-brain coordination is a group of musicians, each playing different notes yet achieving a harmonious outcome by following the same tempo—without, of course, a conductor (see Kelso and Engstrom, 2006, p.93). We hypothesize that this mechanism of inter-brain coordination springs from the very weak coupling engendered by perceptual flows (i.e., weaker than connectivity-based information flows within brains). We further speculate that this weak coupling promotes the emergence of complexity in social interaction (Tognoli et al., 2011).

## Relation to Other Work

A vast literature has emerged in the previous decade regarding neural oscillations involved in social behaviors (reviews in Hari, 2006; Perry et al., 2010; Konvalinka and Roepstorff, 2012; Keller et al., 2014). This literature grew -in the wake of the discovery of the mirror neuron system—with much emphasis on mu rhythm’s suppression during action observation and related social activities (e.g., Cochin et al., 1999; Babiloni et al., 2002; Muthukumaraswamy et al., 2004; Oberman et al., 2007; Cheng et al., 2008; Arnstein et al., 2011; Perry et al., 2011; Woodruff et al., 2011; Derix et al., 2012; Dumas et al., 2012; Lachat et al., 2012; Liao et al., 2012; Moore et al., 2012; Naeem et al., 2012; Vanderwert et al., 2013; Hogeveen et al., 2014; Sebastiani et al., 2014; Fitzpatrick et al., 2015; Moreno et al., 2015, to cite a few). The multiple designations given by different scientists to identical rhythms (e.g., central alpha and mu) and the identical name given to distinct neural activity (e.g., alpha used to designate the parieto-occipital rhythm as well as many other oscillatory activities) are obstacles to advances in the field. Our view is that progress toward understanding the relationship between neural oscillations and (social) function will emerge after a standardized taxonomy of EEG rhythms is in place to facilitate inter-study comparisons; the names that we give to neuromarkers represent an effort to organize our own findings with this goal in mind.

We can classify the foregoing literature depending on the methodology and its ability to resolve spatially and functionally specific neural oscillations (Table 3). Many of the earlier studies (type I), and still some today, used power in predetermined frequency bands at electrodes of interest. For instance, mu can be analyzed at electrodes C3 and C4 in the alpha band or one of its subdivisions. This approach incurs a substantial risk that the results are driven by another rhythmic activity than the one that is assumed (for instance, some unpublished analyses in our laboratory suggest that during social tasks such as action observation with college students as subjects, the specific contribution of mu to power at electrodes C3 and C4 in the complete “alpha” band varies from 13–23%, and is commonly dwarfed by parieto-occipital alpha whose large amplitude attenuates slowly across space. This heterogeneity is in line with others’ findings (Braadbaart et al, 2013) that power in the mu band at electrode C3 negatively modulates the BOLD signal from a constellation of brain areas within and beyond the mirror neuron system. Other studies (type II) use scalp signal and canonical frequency bands, but provide contextual information about the power’s spatial distribution exhibiting peaks at the expected location for a rhythm of interest. Due to suboptimal frequency boundaries, the risk of contamination in these studies lies in the aggregation of power from multiple rhythms—though it may be identified somewhat from the complexity of the rhythmic activity’s spatial patterns (with simpler patterns suggestive of lesser bias). Our own approach (type III) also starts with the scalp signal but adapts the frequency band to each rhythm and each subject, in order to further enhance functional specificity. Finally (type IV), efforts to eliminate extraneous variance take the form of source estimations: provided good head models, electrode density and adequate algorithms, such studies attempt to provide information about the involvement of specific brain areas.

**Table 3:** **Neural epistemology**.

	a. Establish existenceof a neuromarker, oscillation, rhythm	b. Assess modulation of power in taskand controlconditions	c. Analyze unbiased coordination dynamics	d. Estimate sourcelocation
Type I: Power in standard frequency bands, at electrodes of interest, sans spatial context
Type II: Power in standard frequency bands, at peak electrodes, with spatial context	✓	✓
Type III: Power in adjusted frequency bands, at peak electrodes, with spatial context	✓	✓	✓
Type IV: Power in standard frequency bands, in source space	✓	✓		✓

*Summary of approaches to assess oscillatory power in studies of social functions, and their appropriateness to different scientific questions. See details in text*.

To date, we are not aware of source estimation studies that tuned frequency boundaries (as here) in order to further eliminate extraneous variance. Some of our work strongly suggests that at the macroscale of EEG signals, the brain’s spatiotemporal patterns are intermittent rather than continuously modulated in amplitude (Tognoli and Kelso, 2009, 2014; see also Figures 3, 7), although it is highly probable that continuous activity underlies the smaller scales (Figure 5 in Tognoli and Kelso, 2014). Under this hypothesis, the common finding of type IV studies that brain dynamics is continuously modulated (as opposed to a discrete succession of onsets and offsets) appears unlikely. A further possibility is that when a main spatio-temporal pattern recedes, other sources fill-in and contaminate the former source dynamics. With the typically complex brain activities involved in social behavior this problem is aggravated because of the enhanced likelihood that task-related neuromarkers overlap. In our view, an ideal approach, yet to be realized, combines type III and type IV studies in that order.

With the above considerations in mind, and with due caution regarding direct comparisons between topographies obtained using different EEG montages and methods, in the following we attempt to map some of our neuromarker findings with the literature (question a in Table 3), for those studies in which we found sufficient spatial and spectral information to do so. Resolution of questions b and c (power modulation, brain patterns’ spatiotemporal dynamics) would require replications or reanalysis of the respective studies due to the unforeseen effects of extraneous variance—an important issue but well beyond the scope of this work.

Our finding of alpha as an important neuromarker of social function echoes other studies that suggested its importance for the integration of sensory information into social perception, social behavior and (joint) attention (Babiloni et al., 2002; Perry et al., 2010, 2011; Lachat et al., 2012; and with MEG: Sebastiani et al., 2014). The latter work is of both a synchronic and diachronic nature and is in agreement with our findings. We also observed a medial mu in our synchronic studies of social coordination (Tognoli et al., 2007a, 2011). This rhythm distributed its power broadly in frequency and in space, with a mellow peak in the low part of the 10 Hz range over the midline at the level of electrode FCz; power was attenuated during social interactions irrespective of how people coordinated. This rhythm’s frequency and topography might relate to the finding by Moreno et al. (2015), of a central mu that is suppressed during reading of action language (as opposed to abstract language)—although it is difficult to classify this study with respect to synchronic or diachronic behavior since it is a study of single subjects.

In diachronic behaviors such as action observation and delayed imitation, we observed the occurrence of two other mu rhythms with a clear lateralization and a slightly faster frequency than mu medial. The mu rhythms we found perhaps reflect their historical definition since they were located above the Rolandic fissure. Our findings seem to map in a congenial way with a large number of studies of action observation, execution, imagination and imitation (Babiloni et al., 2002; Muthukumaraswamy et al., 2004; Cheng et al., 2008; Perry et al., 2010, 2011; Arnstein et al., 2011; Avanzini et al., 2012; Lachat et al., 2012; Moore et al., 2012; Braadbaart et al, 2013; Sebastiani et al., 2014).

A further finding in our diachronic studies, a parietal rhythm, nu, appeared to be suppressed during action execution, but comparatively less so when the action was being observed. It is possible that this rhythm concurs with findings of parietal mu modulation (Babiloni et al., 2002; Avanzini et al., 2012). Though less obvious because of its smaller spectral footprint and amplitude, we hypothesize that the nu rhythm may well be present in other studies, yet elude detection due to methodological factors. In the same manner, the other neuromarkers that were discovered in our synchronic and diachronic studies (phi and kappa respectively) were of modest size as compared to alpha and mu, and may not make themselves apparent unless specifically parsed as described in Section “The neuromarker framework: finding local oscillations”.

## Summary and Conclusions

Social neuroscience is a young discipline. Accordingly in this review we have focused more on finding the right questions than providing definitive answers about the functional and dynamic architecture of social brains. Our aim was to establish a comprehensive framework to study the dynamics of brains as they evolve through successive phases of social interaction. Such a dynamical framework seems necessary if we are to understand normal and pathological social function. Using a novel set of techniques, a number of neuro-functional signatures of social behavior were uncovered, each with a specific topography and frequency, and each based on continuous brain dynamics requiring high temporal precision. We have drafted some tentative directions for functional inference on newly discovered and lesser known neuromarkers, keeping in mind that more information is needed to converge upon solid interpretations.

Social behavior is grounded in perception~action coupling, a fundamental organizing principle of intentional living beings (see also Prinz, 1997): in the absence of action from an individual, there is no information flow to another’s brain. Without sensitivity to this information by the receiver’s perceptual system, there can be no effective social interaction. We have stressed the primacy of information flows across individuals, and we have shown their fundamental importance for attention—an aspect, perhaps, that has received insufficient scrutiny in social neuroscience.

We examined interpersonal perception-action coupling from the standpoint of the relative phase between individuals (simultaneous or diachronic action~perception). Of course, what we describe as synchronic and diachronic behaviors are limit-cases of a continuum of social circumstances that varies systematically with the phase of each participant’s action. Yet, heuristically, this taxonomy proved useful in revealing little overlap between respective neuromarker landscapes. At several levels of temporal precision (e.g., across tasks, through average activity over trials, and through instantaneous activity), we emphasized the complex reorganization of endogenous brain networks leading to different phases and facets of social behavior.

From the multiplicity of functional processes, and from our findings that the underlying neuromarkers tend not to arise simultaneously, we have begun to enquire about their engagement and disengagement over the course of social interaction, a step that we hope will help refine functional (dynamical) modeling. In our opinion, much work remains to unravel the neural choreography of the cognitive, affective and behavioral processes that participate in social behavior and to embed them in theoretical/computational models of social brain function. Keys to future progress lie with studies of neuromarker coordination in social settings, which, as in other systems such as bimanual and sensorimotor coordination, will lead to modeling the neuro-functional architecture of the social brain.

Already, the present dynamical approach to social brains has revealed some unique coordinative mechanisms that truly relate to social neuroscience (as opposed to a generalization of cognitive neuroscience to social tasks). That is, with the help of the dynamical framework presented in Section “The neuromarker framework: brain coordination dynamics”, we have encountered preliminary evidence that spatiotemporal patterns of brain activity tend to switch in synchrony in pairs of subjects that establish or dissolve behavioral coordination (Benites et al., 2010; Fuchs et al., 2010). These synchronized transitions happened even as one subject’s neural activity differed from that of the other. This finding reveals once more that the interplay of integrative and segregative tendencies within (and now between) brains is a powerful mechanism of nature to enhance system complexity (Kelso, 1995; Edelman, 1999; Sporns, 2003; Kelso and Tognoli, 2007). It is at the level of multiple brains and multiple behaviors, within a complex systems framework, that dynamical models of social function are likely to be ultimately formulated.

## Conflict of Interest Statement

The authors declare that the research was conducted in the absence of any commercial or financial relationships that could be construed as a potential conflict of interest.
