# Supplementary material for: The coordination dynamics of social neuromarkers
Source: Front Hum Neurosci. 2015 Oct 20;9:563. doi: 10.3389/fnhum.2015.00563 (PMC4617382; doi:10.3389/fnhum.2015.00563)
Supplement: Supplementary file 1 [file Presentation_1.pdf]

Supplementary material

Table of contents

S1: Task outline for study 1: Spontaneous social coordination – a synchronic social behavior

S2: Task outline for study 2: Intentional social coordination – a synchronic social behavior

S3: Task outline for study 3: Action observation and delayed imitation – two diachronic social behaviors

S4: Methodology for quantification of neuromarker dynamics

S1 - Task outline for study 1: Spontaneous social coordination – a synchronic social behavior

Sixteen subjects participated in a study of spontaneous social coordination, which consisted of 36 trials each lasting 1 minute (Fig. S1). Subjects were instructed to maintain a comfortable frequency at all times during a trial, and hold fixation of their gaze on a small dot superimposed on a liquid crystal screen placed between the subjects. The dot was positioned to coincide with the location of the other participant's hand. The liquid crystal screen controlled vision of the other participant: it was transparent during the central period (Fig. S1-B), in between two control periods when participants could not see each other.

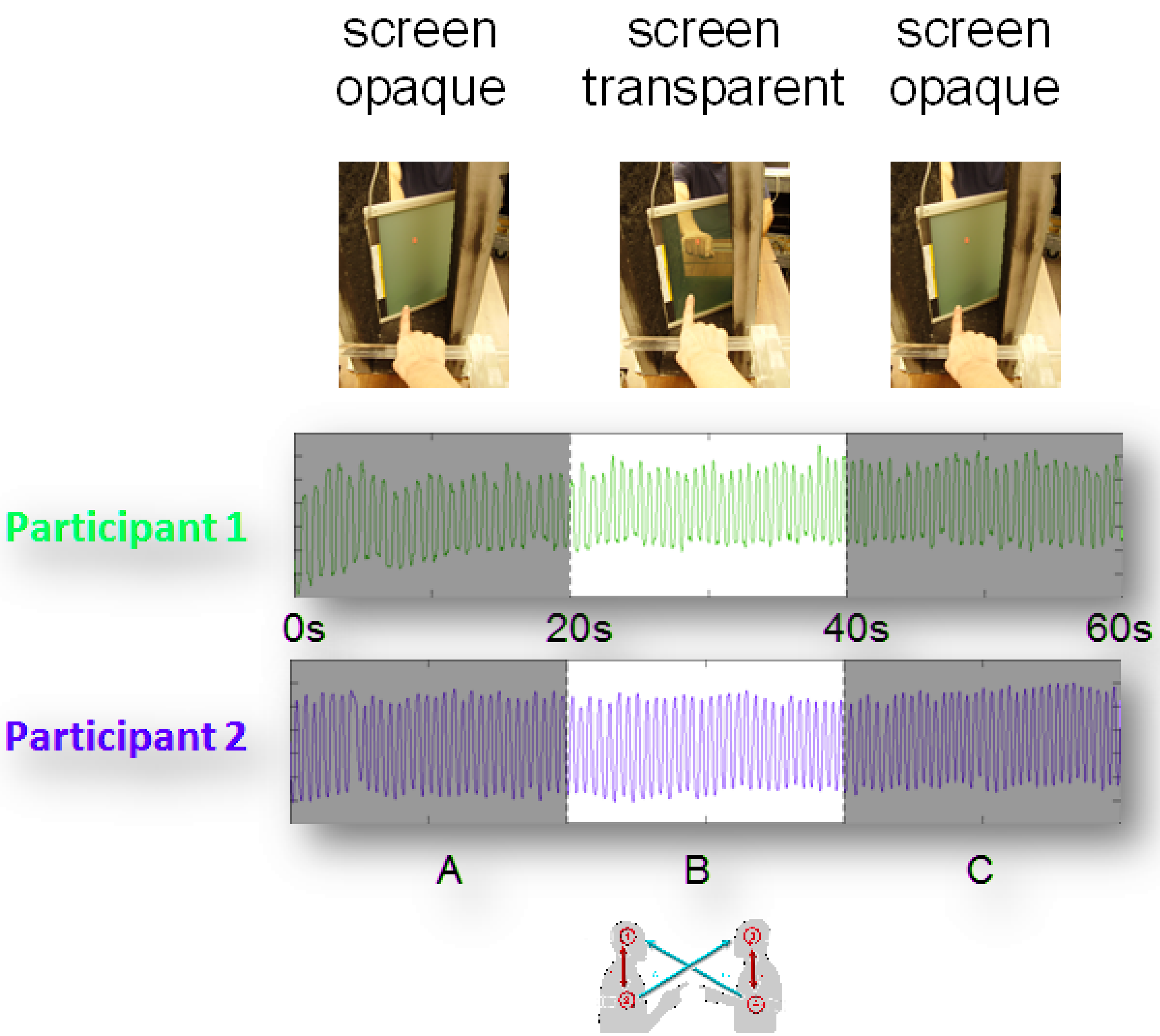

Figure S.1: In study 1, each trial is composed of 3 periods, (A) opaque screen (top left) prevents subjects seeing each other: subjects move their right index finger rhythmically at their intrinsic frequencies (respective movement trajectories in green and purple); (B) screen turns transparent and both participants continue their behavior while watching the other's, under the instruction to move at a comfortable frequency; (C) the screen returns to opaque and participants continue to move without seeing each other. During the central period B, information flows reciprocally within and between subjects, as shown in the little vignette (bottom), see also figure 1, main document.

S2 - Task outline for study 2: Intentional social coordination – a synchronic social behavior

Twenty-four subjects participated in a study of intentional social coordination, which consisted of 60 trials each lasting 40 sec (equivalent to periods A and B from figure S1). The apparatus was similar to that of study 1 (see above). Subjects were instructed to move their hand at a comfortable pace for the first 20 sec. In condition ‘inphase’ (20 trials), subjects were asked to synchronize their movement when the screen became transparent. In condition ‘antiphase’ (20 trials), at the onset of visual contact subjects were asked to coordinate their motion such that the peak flexion of one participant coincided with the peak extension of the other. In condition ‘intrinsic’, subjects were asked to maintain a comfortable frequency, and to try not to synchronize with their partners when vision was allowed.

S3 - Task outline for study 3: Action observation and delayed imitation – two diachronic social behaviors

Fourteen subjects participated in study 3. They were assigned the roles of leader (subject providing a model behavior for the other participant to imitate) or follower (observing the leader's movement and reproducing it after a delay) – a role that they kept for the entire experiment composed of 40 identical trials. In each trial (Fig. S2), subjects were prompted by auditory signals over a succession of 5 periods (A-E). Subjects started moving at their comfortable frequency without view of each other (A). After 8 seconds, the liquid crystal screen interposed between them turned transparent: leader continued to move while follower observed (B). Over the next period (C) both participants rested with their hand immobile. In period (D), the follower reproduced the movement previously observed from the leader. In a final period (E), both participants rested again.

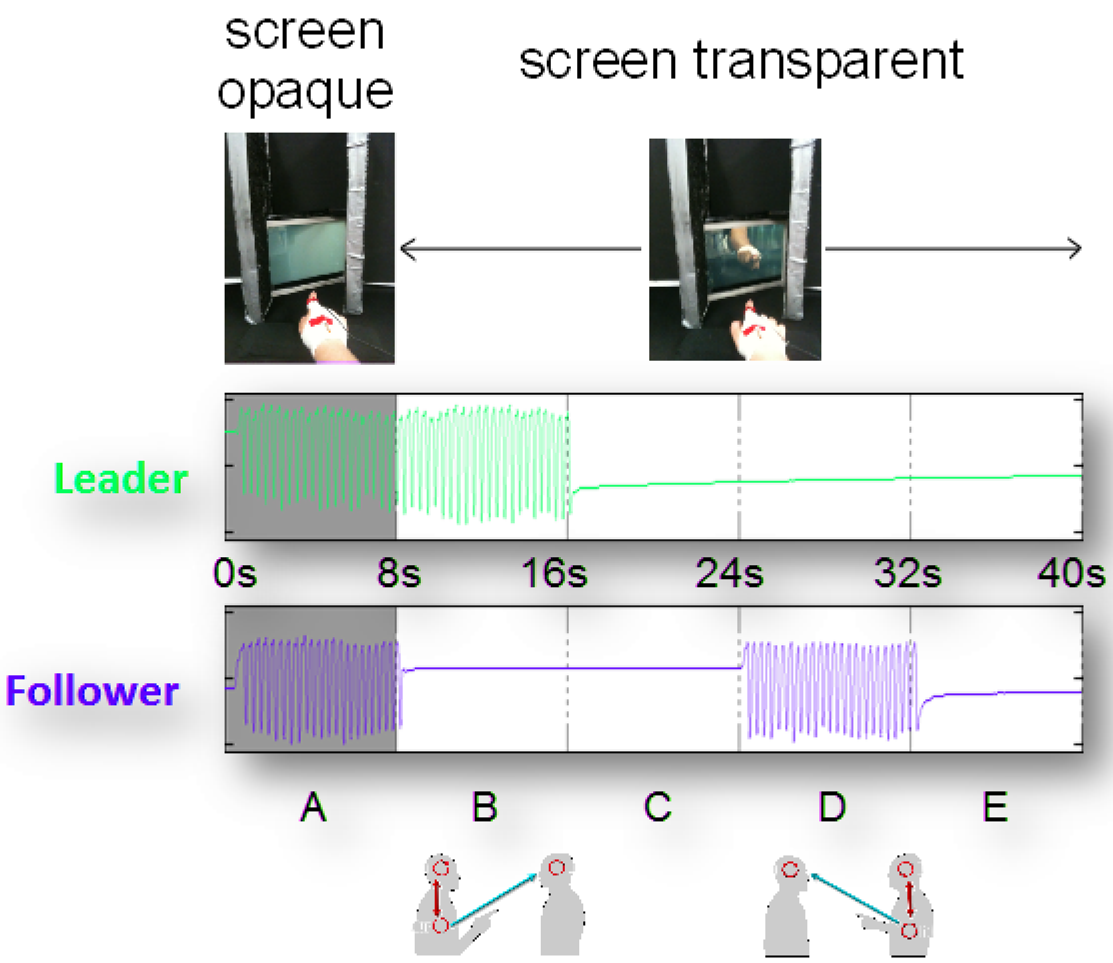

Figure S.2: In study 3, each trial is composed of 5 periods: (A) opaque screen (top left) prevents subjects seeing each other; subjects move their right index finger rhythmically at their intrinsic frequencies (respective movement trajectories in green and purple); (B) screen turns transparent to the end of the trial (top right): one participant, randomly assigned the role of leader continues to move while the partner observes; (C) both participants rest; (D) the participant assigned the role of follower imitates the leaders' previously observed movement; and (E) both participants rest again. When allowed to perceive each other (periods B-E), subjects experience two periods of diachronic behavior in B and D respectively. During periods B and D, information flows unidirectionally between subjects, as shown in the little vignette (bottom), see also figure 1, main document.

S4 - Methodology for quantification of neuromarkers dynamics

In order to capture the neurophysiological mechanisms of social tasks, we attempt a translation between the brains' momentary patterns and specific aspects of social function. Fig. S3 illustrates the steps leading to this end, from parsing neuromarkers (A), with concern for subjects' individual variability (B); to exploring the coordination (C) while avoiding false interpretation of quantitative measures; and analyzing the dynamics (D), either with faster approaches to the neuromarker's probable temporal course of oscillatory power (top) or with an expensive, but accurate analysis of brain patterns' temporal organization (bottom).

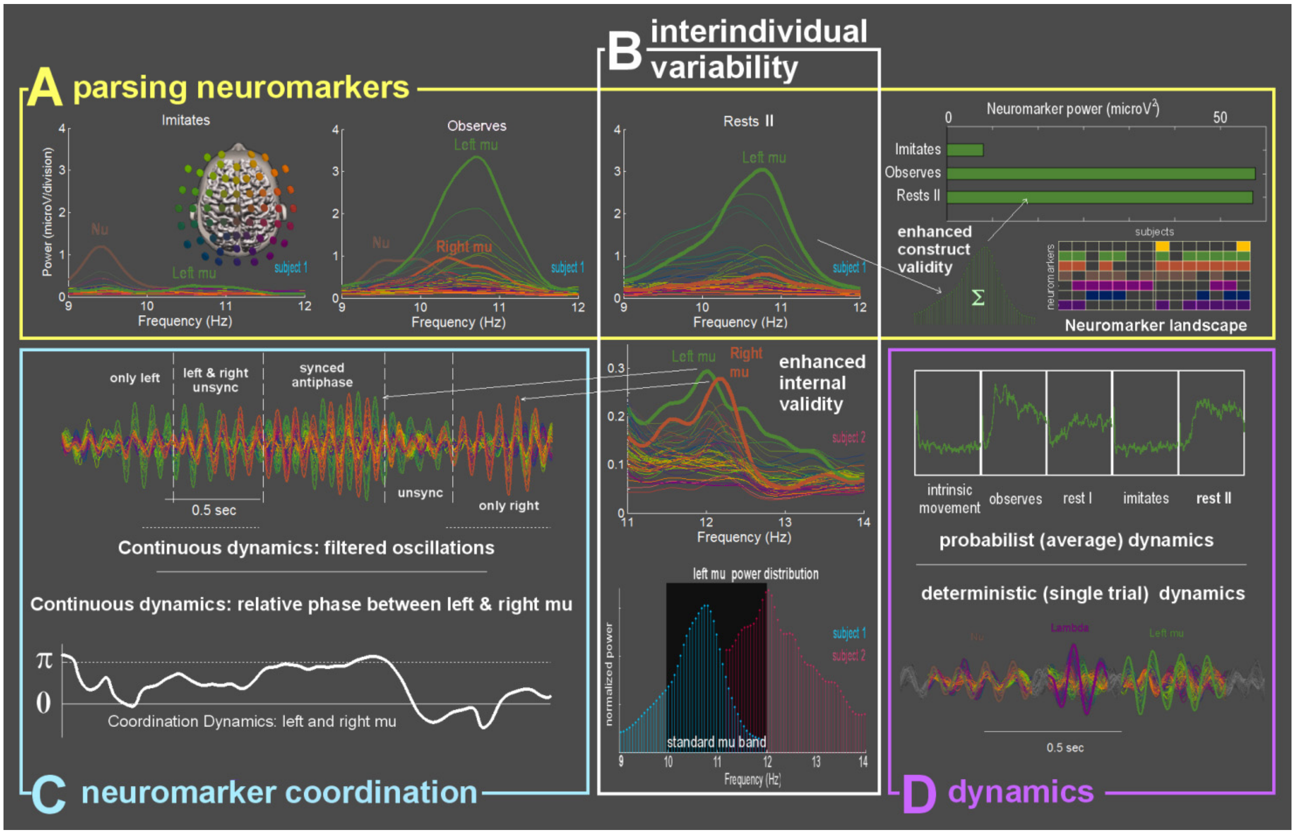

Figure S3: Overview of the steps involved in the study of neuromarker coordination dynamics, illustrated via data from a diachronic study of action observation and delayed imitation (Suutari et al., 2010). In (A), (yellow frame), the power spectrum is examined in each condition to parse the neuromarkers, compute their power (upper right), and derive a neuromarker landscape (lower right). (B, white frame) shows how variable are the neuromarkers' frequency bands from subject to subject. Note the shift of left mu between subject 1 (top) to subject 2 (middle), and observe both subjects' mismatch with standard upper 'alpha' band (bottom). (C, blue frame) illustrates how continuous dynamics guides the interpretation of neuromarker coordination. On top, a sample epoch shows the intermittent coordination between left and right mu rhythms. Their continuous relative phase (bottom), or time-aggregated measures of their phase coupling, would provide an inaccurate picture of phase-locking, since they spend more time without coincidence (first and last pattern) or without synchronization (second and fourth pattern), than synchronized (e.g. central epoch labelled 'synced antiphase'). (D) shows two kinds of dynamical analysis. A probabilistic one is based on the power dynamics (wavelet analysis) at the electrode exhibiting peak power for a neuromarker, and in bins corresponding to the neuromarker's spectral footprint (top). A deterministic one consists in analyzing the patterns' coordination instantaneously and sequentially.
